# Functional analysis and comparative genomics of *Rahnella perminowiae* S11P1 and *Variovorax* sp. S12S4, two plant growth-promoting rhizobacteria isolated from *Crocus sativus* L. (saffron) rhizosphere

**DOI:** 10.1186/s12864-024-10088-6

**Published:** 2024-03-18

**Authors:** Rahma ZOUAGUI, Houda ZOUAGUI, Jamal AURAG, Azeddine IBRAHIMI, Laila SBABOU

**Affiliations:** 1 Laboratory of Microbiology and Molecular Biology, Faculty of Sciences, Mohammed V University in Rabat, Rabat, Morocco; 2 Biotechnology Lab (MedBiotech), Bioinova Research Center, Rabat Medical & Pharmacy School, Mohammed V University in Rabat, Rabat, Morocco

**Keywords:** Genome mining, Core-based phylogeny, Comparative genomics, Plant growth promoting rhizobacteria (PGPR), *Rahnella perminowiae*, *Variovorax* Sp

## Abstract

**Background:**

*Rahnella perminowiae* S11P1 and *Variovorax* sp. S12S4 are two plant growth-promoting rhizobacteria that were previously isolated from the rhizosphere of *Crocus sativus* L. (saffron), and have demonstrated interesting PGP activities and promising results when used as inoculants in field trials. To further elucidate the molecular mechanisms underlying their beneficial effects on plant growth, comprehensive genome mining of S11P1 and S12S4 and comparative genomic analysis with closely related strains were conducted.

**Results:**

Functional annotation of the two strains predicted a large number of genes involved in auxin and siderophore production, nitrogen fixation, sulfur metabolism, organic acid biosynthesis, pyrroloquinoline quinone production, 1-aminocyclopropane-1-carboxylate (ACC) deaminase activity, volatile organic compounds production, and polyamine biosynthesis. In addition, numerous genes implicated in plant-bacteria interactions, such as those involved in chemotaxis and quorum sensing, were predicted. Moreover, the two strains carried genes involved in bacterial fitness under abiotic stress conditions. Comparative genomic analysis revealed an open pan-genomic structure for the two strains. COG annotation showed that higher fractions of core and accessory genes were involved in the metabolism and transport of carbohydrates and amino acids, suggesting the metabolic versatility of the two strains as effective rhizosphere colonizers. Furthermore, this study reports the first comparison of Multilocus sequence analysis (MLSA) and core-based phylogenies of the *Rahnella* and *Variovorax* genera.

**Conclusions:**

The present study unveils the molecular mechanisms underlying plant growth promotion and biocontrol activity of S11P1 and S12S4, and provides a basis for their further biotechnological application in agriculture.

**Supplementary Information:**

The online version contains supplementary material available at 10.1186/s12864-024-10088-6.

## Background

According to the latest report of the Food and Agriculture Organization (FAO), one out of five people have faced hunger in Africa, and 9% of the Asian total population was undernourished in 2021 [1]. In response to the growing demand for food, chemical fertilizers have been extensively used to enhance soil fertility and boost crop productivity. However, the prolonged use of chemical fertilizers has affected soil ecology and raised serious environmental concerns [2]. Moreover, crops encounter various abiotic stresses such as salt, drought, heavy metals, and extreme temperatures, which affect crop productivity and severely threaten global food security [3]. The concept of plant growth-promoting rhizobacteria (PGPR) was proposed by Kloepper and Schroth in 1978 [4], and has received growing attention over the last three decades [5]. PGPR are free-living soil microorganisms that inhabit the rhizosphere and are capable of enhancing plant growth and resistance in normal and stressed soils [3]. PGPR exert beneficial effects through both direct and indirect mechanisms. The direct mechanisms include phytohormone production, phosphate stabilization, and nitrogen fixation. Indirect mechanisms involve the production of antimicrobial compounds, induction of systemic resistance, and competition for nutrients in the rhizosphere [5]. Plant growth-promoting rhizobacteria associated with *Crocus sativus* L. (saffron) have been extensively explored using cultivation-dependent methods [6–14], and metagenomic approaches [15–17]. However, despite the large number of PGPR strains secluded from saffron plant, few studies have investigated the genomic basis underlying their plant-beneficial activities. Indeed, only four studies have explored the plant growth-promoting activities of rhizobacteria associated with the saffron plant from a genomic perspective, focusing exclusively on strains within the *Bacillus* and *Pseudomonas* genera [6, 7, 18, 19].

Many species of *Rahnella* and *Variovorax* genera have been described as plant growth promoters and effective biocontrol agents against numerous soil-borne pathogens [20–23]. *Rahnella* is a Gram-negative, facultative anaerobic, rod-shaped bacterium that belongs to the *Yersiniaceae* family [24]. *Rahnella* species have been isolated from various environments, including soil, phyllosphere, water, seeds, food, and clinical samples [25–29]. The genus *Rahnella* includes 13 recognized species [30], among which *Rahnella perminowiae* and three other new species were recently proposed in January 2022 [31]. According to the literature, many *R. aquatilis* strains have been reported to improve poplar and Masson pine growth [32]; increase plant height, root length, and aboveground part weight of Chinese cabbage [33]; inhibit the mycelial growth of *Colletotrichum gloeosporioides* by producing volatile organic compounds (VOCs) [20]; and inhibit the crown gall disease propagation by producing antimicrobial substances [21]. Furthermore, many studies have demonstrated the ecological fitness and strong environmental adaptability of *Rahnella* strains owing to their tolerance to acids, salts, selenium, antibiotics, and heavy metals [34–37].

*Variovorax* species are gram-negative, facultative, anaerobic bacteria belonging to the *Comamonadaceae* family [38]. At the time of writing, the genus *Variovorax* comprised eight species with valid names [30], many of which have been isolated from soil [39–41], and a few species have been isolated from other environments, such as plants [42] and sewage [43]. Some *Variovorax* species have also been described as PGPR. For example, *V*. *paradoxus* 5 C-2 promotes plant growth in arid soil through its 1-aminocyclopropane-1-carboxylate (ACC) deaminase activity [22]. Similarly, *Variovorax* sp. PMC12 enhances tomato defense against the causal agent of the bacterial wilt disease *Ralstonia solanacearum* under high salinity and low-temperature conditions [23]. In addition, several reports have emphasized the high metabolic versatility of *Variovorax* strains and their ability to degrade biogenic compounds and anthropogenic contaminants such as pesticides and aromatic compounds [44–47]. Moreover, *V. paradoxus* is known to be tolerant to a number of heavy metals such as arsenate, chromate, cadmium, and mercury [48]. Owing to these metabolic features, *Variovorax* species are recognized as strong competitors in the rhizosphere, even in hostile habitats [22].

To date, most studies of the plant growth-promoting activities of *Rahnella* and *Variovorax* species have focused on *R. aquatilis* and *V. paradoxus*. Moreover, only one report has described the general physiological, biochemical, and phylogenetic characteristics of the recently proposed species *R. perminowiae* [31]. Consequently, information regarding its genetic characteristics, taxonomic position, and potential biological activity remains limited.

*R. perminowiae* S11P1 and *Variovorax* sp. S12S4, (hereafter referred to as S11P1 and S12S4, respectively), are two plant growth promoting rhizobacteria that have been previously isolated from the rhizosphere of saffron plant, and were screened for auxin and siderophore production, phosphate solubilization, nitrogen fixation, ACC desamine activity, and antagonism against *Fusarium oxysporum* [13]. These two strains, have also demonstrated promising results when tested as consortia under field conditions [13].

The present study aims to investigate the molecular basis of the plant growth promotion activities, biocontrol potential, and lifestyle strategies of S11P1 and S12S4. For this purpose, a polyphasic approach integrating phylogenetic, phylogenomic, genomic, and comparative genomics was employed. To the best of our knowledge, this is the first genomic analysis of *Rahnella perminowiae* and a potential novel species of the *Variovorax* genus isolated from the rhizosphere of saffron plant. The results of this study provide in-depth insights into the mechanisms of the plant growth promotion capabilities of S11P1 and S12S4, thus supporting their practical application as biofertilizers and biostimulants.

## Results

### Genomes characteristics

The raw reads were assembled in 7 and 2 contigs for S11P1 and S12S4, respectively, with a genome size of 5.9 Mbp for both strains. The S11P1 and S12S4 genomes comprised a total of 5401 and 5719 coding sequence, with GC content of 51.4% and 65.7%, respectively. Other genome assemblies and annotation metrics have been described in our previous genome announcement [49].

### Taxonomic position of S11P1 and S12S4

**Fig. 1:**
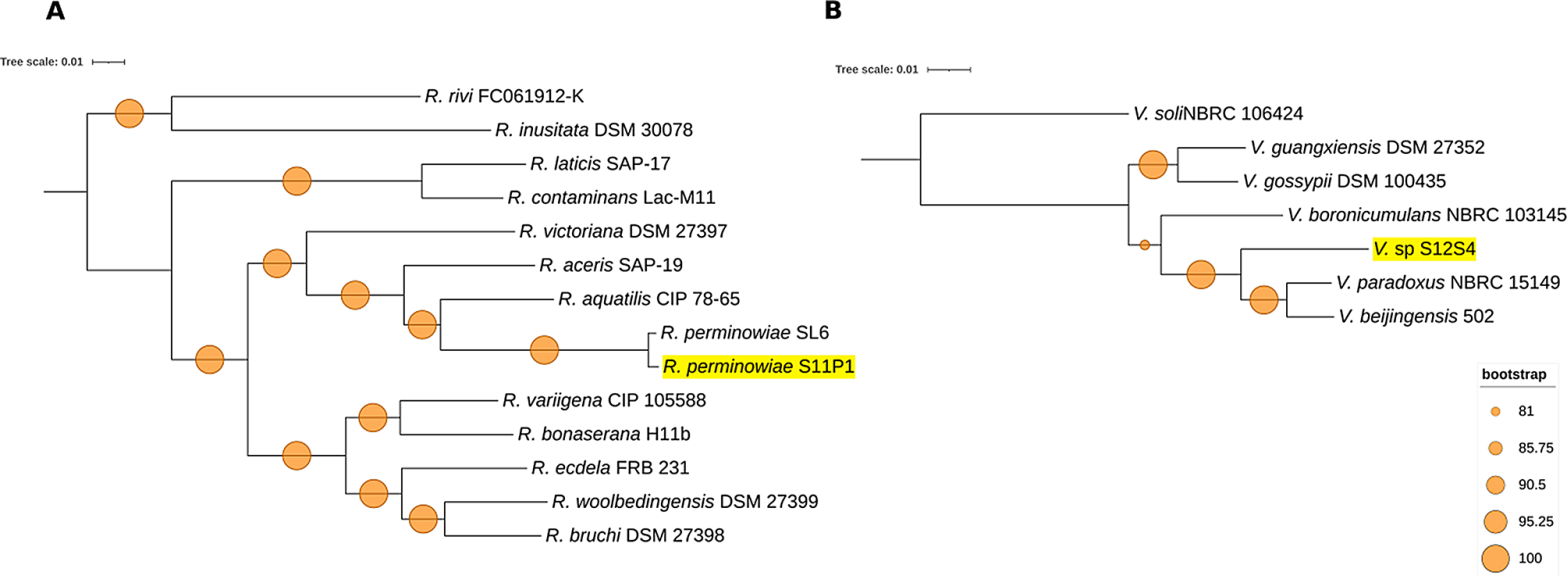
Midpoint rooted phylogenomic trees of the *Rahnella* and *Variovorax* genera. The maximum-likelihood trees of *Rahnella perminowiae* S11P1 (**A**) and *Variovorax* sp. S12S4 (**B**), with corresponding genera-type strains, were constructed based on 1469 and 77 concatenated core-genome sequences, respectively

To define the taxonomic positions of S11P1 and S12S4, whole-genome comparisons with all available type-strain genomes were performed using the TYGS server. The comparisons showed that strains S11P1 and S12S4 belonged to the genera *Rahnella* and *Variovorax*, respectively. Furthermore, we performed MLSA of four concatenated genes (16 S rRNA, *gyrB*, *rpoD*, and *rpoB*) and phylogenomic analyses based on 1469 and 77 core genes of the *Rahnella* and *Variovorax* genera, respectively. Based on the MLSA and phylogenomic trees, S11P1 was grouped in a single cluster with *R*. *perminowiae* SL6, with a bootstrap support of 100%, whereas S12S4 formed a monophyletic cluster with *V*. *paradoxus* NBRC 15,149 and *V*. *beijingensis* 502 (Fig. 1, Supplementary Fig. S1). Furthermore, S11P1 shared ANI and dDDH values of 99.05% and 91.5%, respectively, with *R*. *perminowiae* SL6, indicating that they belong to the same species. S12S4 demonstrated ANI and dDDH values of 89.4% and 36.4%, respectively, for the best matches against *V*. *beijingensis* 502, suggesting that it could represent a novel bacterial species of the genus *Variovorax* (Supplementary Table S3).

### Genome mining for genes encoding plant growth-promoting traits

**Fig. 2:**
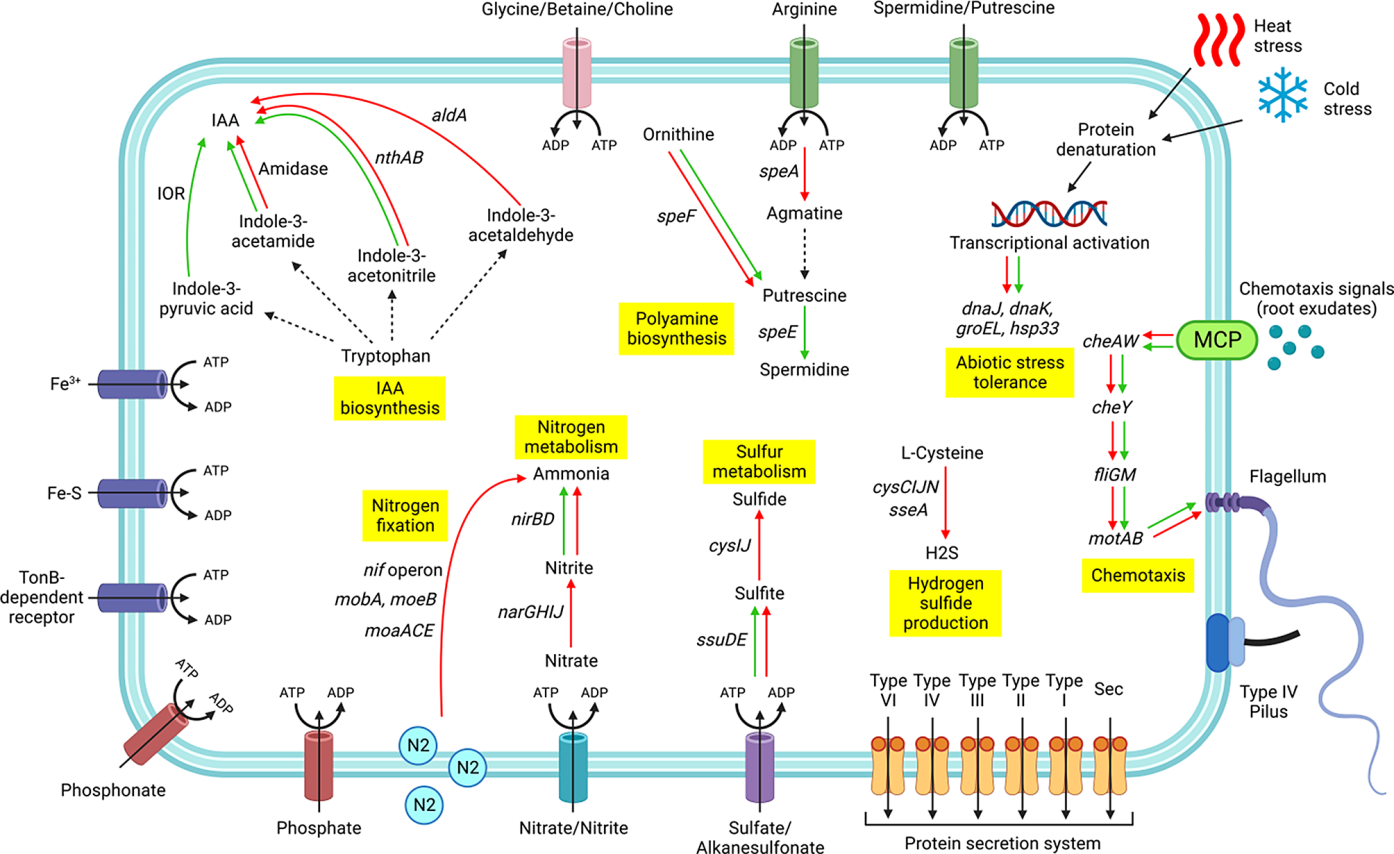
Schematic overview of plant growth promotion and stress tolerance genes predicted for S11P1 and S12S4. The genes are represented with colored arrows: red (S11P1) and green (S12S4). Dashed arrows represent genes missing in both genomes. Complete gene names and descriptions are provided in Supplementary Table S4. The figure was created with BioRender.com

Functional analysis of the S11P1 and S12S4 genomes revealed the presence of several genes involved in multiple PGP functions, including auxin and siderophores biosynthesis, phosphate solubilization, nitrogen and sulfur metabolism, root colonization, biocontrol, and abiotic stress resistance (Fig. 2, Supplementary Table S4).

### Auxin biosynthesis

The S11P1 and S12S4 strains harbored the tryptophan biosynthesis gene cluster *trpABCDS*, which is involved in auxin biosynthesis. The two strains also carried genes encoding amidase, histidinol phosphate transaminase (*hisC*), and nitrile hydratases (*nthAB*). The anthranilate synthase (*trpE*) and indolepyruvate oxidoreductase encoding genes were predicted in the S12S4 genome, while genes encoding tryptophan permease, aldehyde dehydrogenase and ubiquinone-dependent pyruvate dehydrogenase were found the S11P1 genome.

### Siderophores and iron uptake

In terms of siderophore production, the S11P1 and S12S4 genomes harbored genes encoding chorismate synthase (*aroC*), ferrochelatase (*hemH*), isochorismatase (*dhbB*), bacterioferritin (*bfr*), and diaminopimelate decarboxylase (*lysA*). The S11P1 genome contains shikimate kinases (*aroKL*) encoding genes together with others for siderophores production, such as chorismate lyase (*ubiC*) and chorismate mutase (*aroH*). The two strains carried several genes encoding siderophore receptors and iron transporters, such as TonB-dependent siderophore receptor, heme ABC transporter ATP-binding protein, and iron ABC transporter permease. Additional siderophore receptors and transport-related genes were predicted in the S12S4 genome, including iron-sulfur cluster assembly protein (*IscA*), Fe-S cluster assembly scaffold (*IscU*), Fe(3+) ABC transporter substrate-binding protein, 2Fe-2 S iron-sulfur cluster-binding protein, and ferrous iron transporters (*feoAB*).

### Phosphate solubilization

The S11P1 and S12S4 genomes encompassed several genes related to phosphate solubilization and assimilation. The two strains shared the pyrroloquinoline quinone gene cluster (*pqqABCD*), whereas *pqqEF* were found only in S11P1. The two strains carried also the genes encoding inorganic diphosphatase (*ppa*), protein phosphatase (*cheZ*), phosphate response regulators (*phoBHU*), phosphate permease (*pstACS*) and phosphonate transporter (*phnE*), whereas genes encoding phosphate transporter protein (*pstB*), phosphonate, and carbon-phosphorus (C-P) lyases (*phnCDFGHIJKL*) were predicted only in the S11P1 genome.

### Nitrogen and sulfur metabolism

The S11P1 genome encodes the gene cluster *nifABDEJHKLMNQSTUWXZ*, along with genes for nitrate reductase (*narGHIJ*), whereas S12S4 lacks the nitrogenase complex. However, genes encoding nitrite reductases (*nirBD*), nitrogen regulation proteins (*glnGL*), and molybdenum cofactors (*mobA*, *moeB*, and *moaACE*) were identified in both the genomes. Additionally, the two strains were predicted to harbor the genes clusters *cysDEKNQTW* and *ssuBCDE* for sulfur metabolism and sulfate/sulfonate transport, respectively. Genes encoding adenylyl-sulfate kinase (*cysC*), sulfite reductase (*cysIJ*), sulfate/thiosulfate transporter (*CysA*), and 3-mercaptopyruvate sulfurtransferase (*sseA*) were found only in the S11P1 genome, whereas genes encoding sulP inorganic anion transporter and sulfite exporter (*tauD*) were observed in the two genomes.

### Chemotaxis and plant root colonization

Relating to plant-bacteria interactions, both strains were predicted to encode methyl-accepting chemotaxis proteins and chemotaxis proteins (*cheAWY*), along with the gene clusters *fliADEFGHIJKLMNPQRS*, *flgABCDEFGHIJKLM*, *flhABCD*, and *motAB*, which encode the machinery for flagellar biosynthesis and motility. The two genomes encode also plant root colonization and adhesion proteins such as hemolysin proteins, tyrosine recombinase/integrase (*XerCD*), and prepilin peptidase. Additionally, the S11P1 genome harbors a series of quorum sensing-related genes, such as S-ribosylhomocysteine lyase (*luxS*), (4 S)-4-hydroxy-5-phosphonooxypentane-2,3-dione isomerase (*IsrG*), 3-hydroxy-5-phosphonooxypentane-2,4-dione thiolase (*IsrF*), homoserine lactone efflux protein (*rhtB*), and autoinducer transporters (*lsrABCDG)*.

### Biocontrol activity

Functional annotation revealed several genes encoding antimicrobial and antifungal compounds. The two genomes harbored genes for cobalamin (*cobOST*) and phenazine (*phzF*, *lysR*) biosynthesis together with genes encoding the resistance inducer enzyme methionine synthase (*metH*). The S11P1 genome carries numerous antimicrobial and antifungal biosynthesis genes. These include rhamnolipids (*rhlBE*), surfactin (*srfAC*), and colicin biosynthesis proteins. The two strains encode numerous lytic enzymes, including glycosyl hydrolase, serine protease, phospholipase, and protease (*htpX*). Moreover, genes encoding the chitinolytic enzyme beta-N-acetylhexosaminidase (*nagZ*), the cellulolytic enzyme beta-glucosidase (*bglX*), and pitrilysin (*ptrA*) were observed only in the S11P1 genome. Furthermore, genes involved in volatile organic compound (VOCs) production were identified in the two genomes; the acetolactate synthase large and catalytic subunits encoded by *alsS* and *ilvNG*, together with acetolactate decarboxylase (*budA*), were found in the S11P1 genome, whereas the acetolactate synthase small and large subunits (*ilvBN*) were predicted in the S12S4 genome.

### Abiotic stress tolerance

In addition to the plant growth-promoting activities, the two genomes were predicted to encode several genes for abiotic stress management. Both strains carried the cold shock genes *dnaJ*, *dnaK*, *groES*, and *groEL* and the heat shock gene *hslO*. Moreover, genes involved in oxidative stress response, such as glutathione peroxidase (*gpx*), gamma-glutamyltransferase (*ggt*), glutathione transferase (*gstA*), glutaredoxin 3 (*grxC*), and thioredoxins (*trxAC*) have been predicted. In addition, the two genomes encode glycine betaine/L-proline transporters (*proVXW*), which are involved in salt stress management. The S12S4 genome contains the ACC deaminase-encoding gene, whereas S11P1 encodes the *betABIT* gene cluster, which is involved in the transport and accumulation of osmolytes. Genes associated with the biosynthesis and transport of polyamines were found in both genomes. These include adenosylmethionine decarboxylase (*speD*), argininosuccinate lyase (*argH*), and arginine–tRNA ligase (*argS*). Spermidine synthase (*speE*) was predicted in the S12S4 genome, whereas the S11P1 genome carries numerous genes for polyamine biosynthesis and transport, such as agmatine deiminase (*aguA*), N-carbamoylputrescine amidase (*guB*), arginine decarboxylase (*speA*), and putrescine/spermidine transporters (*sapBC*; *potFGHI*).

### Genome mining for biosynthetic gene clusters

Genome mining of S11P1 for bioactive secondary metabolites identified nine biosynthetic gene clusters (BGCs). Among the predicted BGCs, two were identified to contain NRPS-independent siderophores, one each encoding desferrioxamine E and vibrioferrin; one homoserine lactone for microcin E492; one homoserine lactone-phosphonate for N-myristoyl-D-asparagine; one NRPS-T1PKS for crochelin A; one thiopeptide for O-antigen; one RRE-containing for vibrioferrin; one gene cluster for aryl polyenes; and one for betalactone. The predicted gene clusters were compared to those deposited in the MIBiG repository. The BGC encoding desferrioxamine E exhibited 100% similarity to an orthologous BGC encoded by *Pantoea agglomerans*, whereas the BGCs encoding aryl polyenes and vibrioferrin showed 77% and 57% similarity to their corresponding orthologous clusters from *Xenorhabdus doucetiae* and *Azotobacter vinelandii* CA, respectively. The remaining gene clusters displayed less than 27% similarity to known BGCs, including one cluster for betalactone with no matches against the MIBiG repository (Supplementary Table S5). For S12S4, three hybrid gene clusters were predicted, comprising one NRP-metallophore, NRPS, T1PKS encoding variochelin; one arylpolyene, resorcinol encoding lipopolysaccharide; and one T1PKS, NRPS-like, NRPS encoding microsclerodermin. The BGC encoding variochelin exhibited 90% similarity to an orthologous BGC encoded by *V. paradoxus* B4, whereas the BGCs encoding microsclerodermin and lipopolysaccharide displayed 21% and 5% similarity to their corresponding orthologous clusters encoded by *Jahnella* sp. MSr9139 and *Xanthomonas campestris* pv. *campestris*, respectively. Additionally, the S12S4 genome harbors four gene clusters encoding arylpolyene, redox-cofactor, resorcinol, and terpene, with no similarity to any known clusters (Supplementary Table S5).

### Comparative genome analysis

**Fig. 3:**
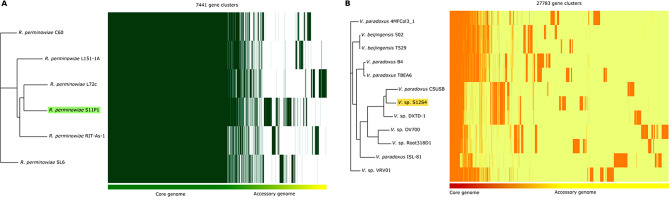
Pan-genome matrices of S11P1 (**A**) and S12S4 (**B**) and their corresponding closest strains. The matrices show gene presence/absence patterns in the core, accessory, and unique fractions of each strain. The maximum-likelihood trees on the left side of the matrices were constructed using core-genome sequences

**Fig. 4:**
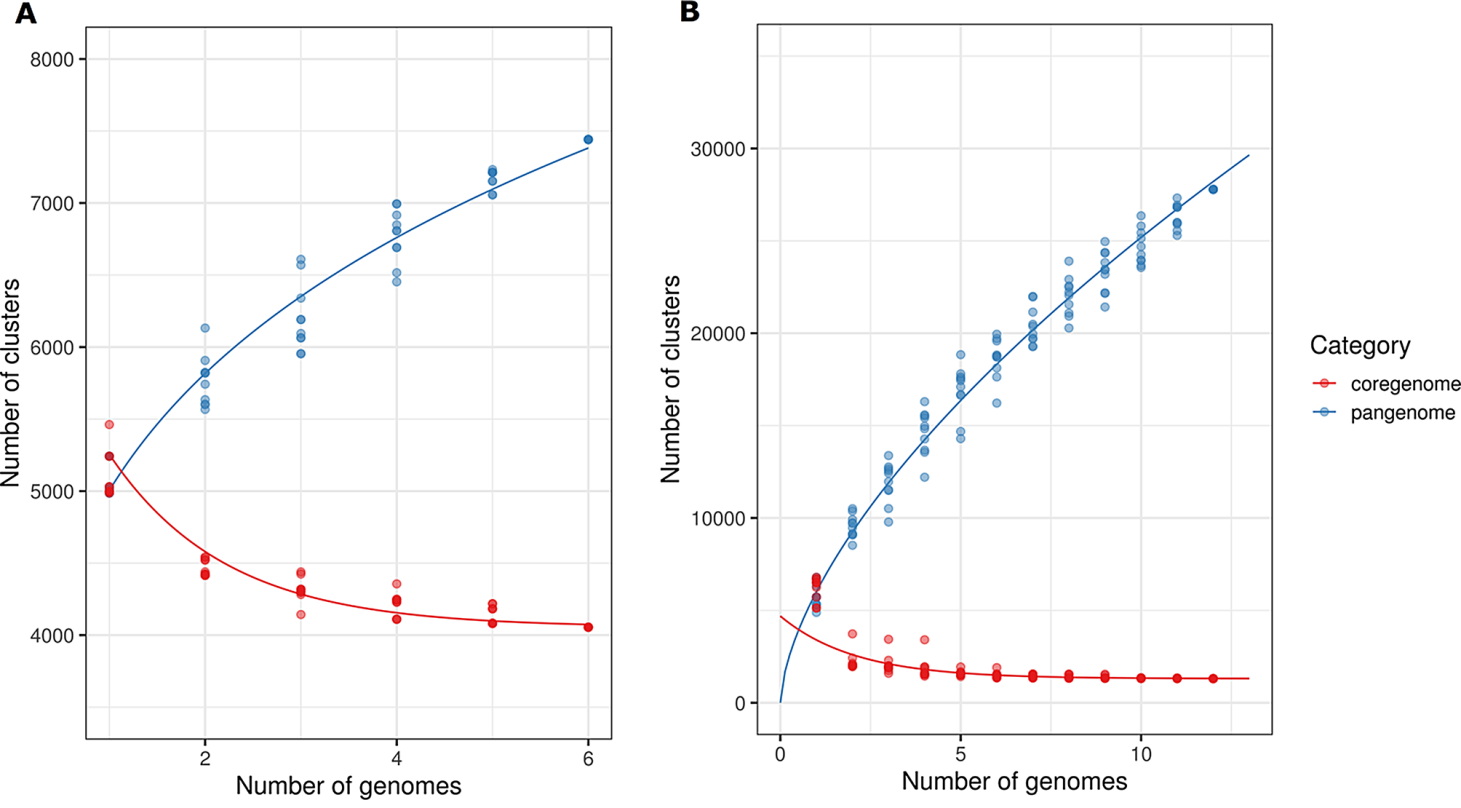
Core and pan-genome cumulative curves of *Rahnella perminowiae* (**A**) and *Variovorax* spp. (**B**). The cumulative curves illustrate an increase in the number of new gene families (pan-genome) and a decrease of shared gene families (core-genome) with the addition of new genomes

**Fig. 5:**
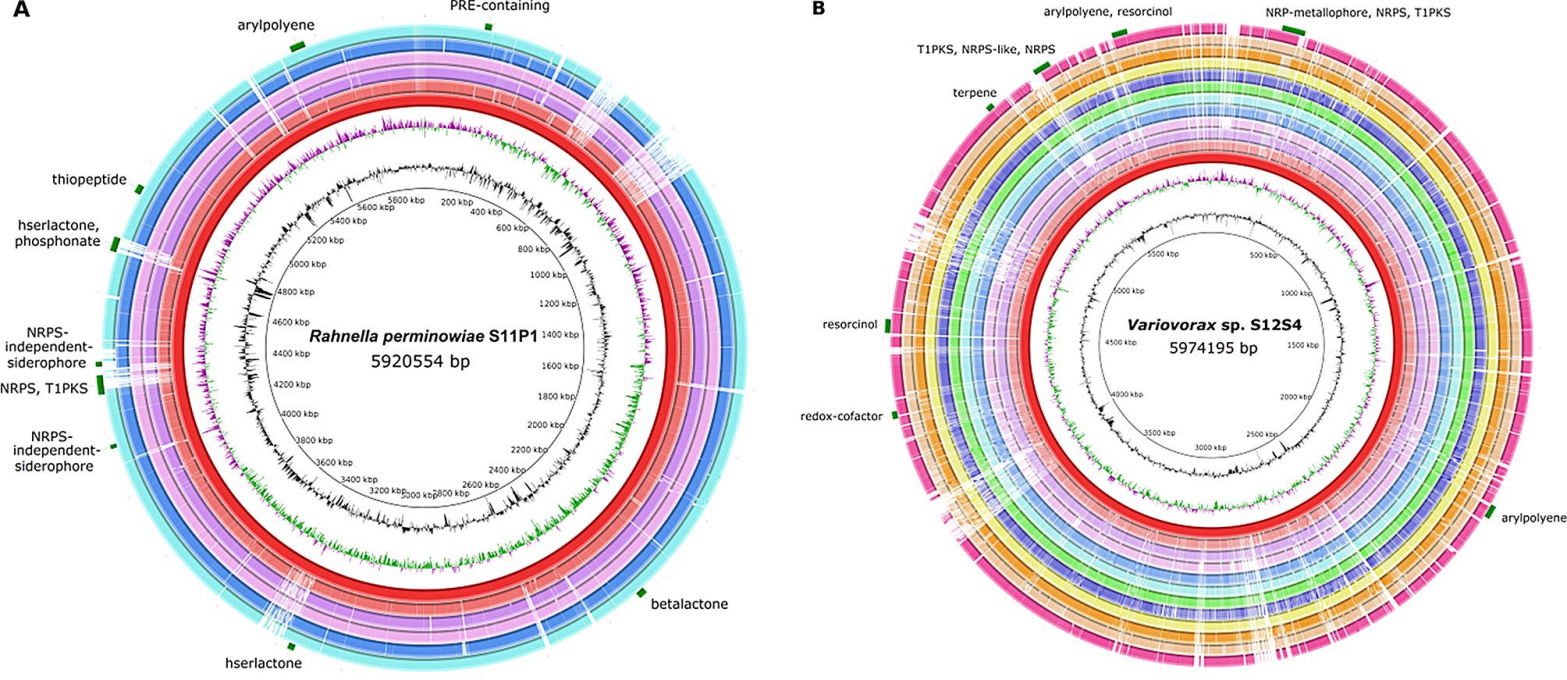
Circular plots of the pan-genomes of S11P1 (**A**) and S1S24 (**B**). The three innermost rings indicate the genome size, GC content, and GC skew of the reference genomes *Rahnella perminowiae* S11P1 (**A**) and *Variovorax* sp. S12S4 (**B**). The outer rings illustrate the BLAST genome comparisons against the corresponding genome references. From the inside to the outside, the rings correspond to the following strains: (**A**) S11P1, RIT-As-1, C60, SL6, L151-1 A, L72C; (**B**) S12S4, TBEA6, T529, B4, VRV01, OV700, 4MFCol3-1, ISL-81, 502, DXTD-1, Root318D1, CSUSB. The green blocks in the outermost ring represent secondary metabolite cluster regions in the reference genomes

**Fig. 6:**
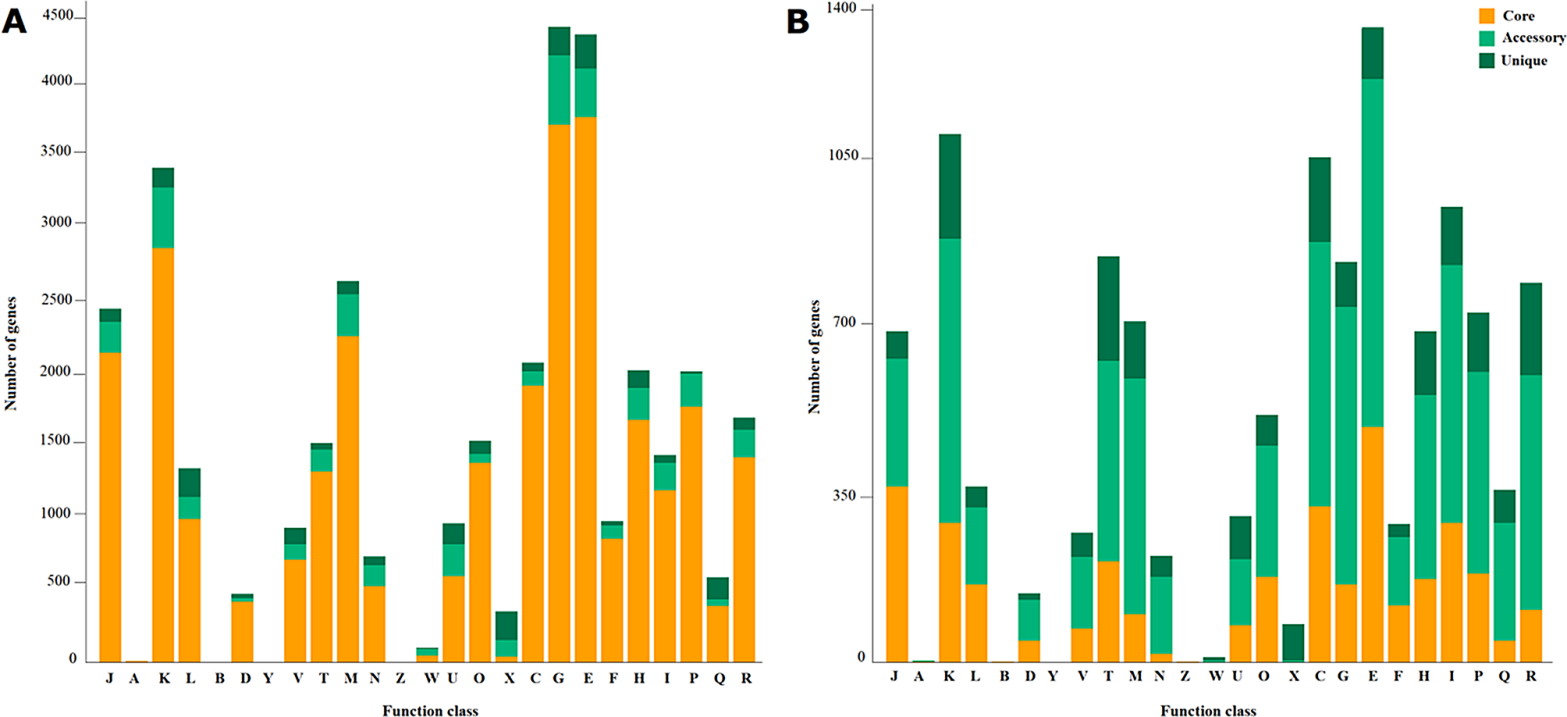
COG distribution among core, accessory, and strain-specific genes of S11P1 (**A**) and S12S4 (**B**). J, Translation, ribosomal structure, and biogenesis; A, RNA processing and modification; K, Transcription; L, Replication, recombination and repair; B, Chromatin structure and dynamics; D, Cell cycle control and mitosis; Y, Nuclear structure; V, Defense mechanisms; T, Signal transduction mechanisms; M, Cell wall/membrane/envelope biogenesis; N, Cell motility; Z, Cytoskeleton; W, Extracellular structures; U, Intracellular trafficking, secretion and vesicular transport; O, Post-translational modification, protein turnover, chaperone functions; X, Mobilome, prophages, transposons; C, Energy production and conversion; G, Carbohydrate metabolism and transport; E, Amino acid metabolism and transport; F, Nucleotide metabolism and transport; H, Coenzyme metabolism and transport; I, Lipid metabolism and transport; P, Inorganic ion transport and metabolism; Q, Secondary metabolites biosynthesis, transport and catabolism; R, General Functional Prediction only

Pan-genome analysis was conducted on S11P1 and S12S4 along with their respective closest neighboring strains. The analysis revealed pan-genome sizes of 7441 and 22,783 gene clusters for S11P1 and S12S4, respectively. Of these, 4055 (54.4%) and 1306 (5.3%) were core genes, 722 (9.7%) and 3023 (13.3%) were accessory, and 668 (8.9%) and 1392 (6.1%) were unique to S11P1 and S12S4 genomes, respectively (Fig. 3). Power law and exponential curve fitting of the pan-genome and core-genome counts showed that the pan-genome of S11P1 is open but may be closed soon, with an α parameter value of 0.54. For S12S4, the pan-genome is open with an α value of 0.27 (Fig. 4). Circular maps depicting the uniqueness of S11P1 and S12S4 across their closest corresponding strains are shown in Fig. 5. Furthermore, the core, accessory, and unique genes of S11P and S12S4 were annotated against the COG database using COGclassifier. The higher proportion of S11P1 genes was assigned to the categories G (11,7%, carbohydrate metabolism and transport), E (11.6%, amino acid metabolism and transport), and K (9.1%, transcription). For S12S4, the most common categories were E (10.3%; amino acid metabolism and transport), K (8.5%; transcription), and C (8.2%; energy production and conversion) (Fig. 6).

## Discussion

Genome-based tools, such as ANI and dDDH, have provided solid criteria for the taxonomical ascription of bacterial species. In this study, the S11P1 strain was identified as *R*. *perminowiae*, whereas S12S4 was predicted to be a potential new *Variovorax* species. MLSA is recognized as a fast, inexpensive, and taxonomically robust option for refining species discrimination. However, selecting conserved genes for MLSA remains challenging for numerous bacterial taxa [50]. Based on our results, the topologies of the MLSA trees were found to be congruent with those of the phylogenomic trees, especially for the clusters comprising S11P1 and S12S4, and their corresponding closely related type strains. Therefore, we propose our MLSA scheme (16 S rRNA, *gyrB*, *rpoD*, and *rpoB*) as an accurate and suitable method for discriminating *Rahnella* and *Variovorax* species.

Indole-3-acetic acid (IAA) production is one of the leading plant growth-promoting mechanisms of beneficial bacteria, and is often regarded as a favorable bacterial trait for biological inoculant design [51]. In bacteria, five different IAA biosynthesis pathways have been suggested using tryptophan as a precursor: indole-3-acetamide (IAM), indole-3-pyruvate (IPyA), indole-3-ethanol (TOL), indole-3-acetonitrile (IAN), and tryptamine (TAM) [52]. Although there is no sufficient evidence for a complete biosynthetic pathway, the two genomes contain enzymes from the IAN, IAM, IpyA, and TAM pathways. Both genomes encode the nitrile hydratase (*nthAB*) enzyme that contributes to the conversion of IAN to IAA in the IAN pathway [53]. Additionally, both genomes encode the amidase enzyme that converts IAM to IAA during the last step of the IAM pathway [53]. However, the first step recruited a tryptophan-2-monooxygenase enzyme (*iaaM*) to convert tryptophan to IAM [54], which was not predicted in either of the genomes. Furthermore, the aldehyde dehydrogenase predicted in the S11P1 genome converted indole-3-acetaldehyde to IAA. This conversion occurs in three of the known IAA pathways: IPyA, TAM, and tryptophan side-chain oxidase (TSO) [53, 55]. The S12S4 genome encodes indolepyruvate ferredoxin oxidoreductases (*ior*), which has recently been reported in a novel alternative route for IAA biosynthesis, in which indole-3-pyruvic acid is converted directly to IAA [56].

Another key role of plant-beneficial bacteria is siderophores production [57]. The two strains contained a variety of genes involved in siderophore production and iron sequestration. These include genes encoding ferrochelatase, isochorismate, bacterioferritin, iron ABC transporter permeases, and TonB receptors. AntiSMASH analysis identified BGCs for vibrioferrin, desferrioxamine E, and crochelin in S11P1, and one BGC for the biosynthesis of Variochelin in S12S4 genome. The presence of such gene clusters, along with siderophore biosynthetic machinery endorses the experimental observation and the field inoculation results of S12S4 [13]. Indeed, it has been observed that inoculation of saffron bulbs with the highly siderophore-producing S12S4 strain increases the fresh and dry weights of leaves and daughter corms, as well as the total chlorophyll content [13].

Phosphate-solubilizing bacteria can convert insoluble phosphate into plant-available forms, which is regarded as a major mechanism of action for PGPR [58]. Based on our genomic analysis, both genomes we predicted to encode phosphatase enzymes. Phosphatases hydrolyze phosphoric acid into phosphorus ions and free OH groups, making it accessible to plants [59]. In addition, the C-P lyase system encoded by the *phn* gene cluster was found to be essential for bacterial degradation of phosphonate [60]. In this study, the entire *phn* gene cluster was observed in the S11P1 genome and the phosphonate transporter *phnE*-encoding gene was predicted in the S12S4 genome. These findings highlight the strong ability of S11P1 and S12S4 to dissolve organic phosphates. Another widely recognized mechanism for solubilizing inorganic phosphate involves the production of organic acids, such as gluconic acid [61]. Gluconic acid production is mainly catalyzed by pyrroloquinoline quinone (*pqq*) and glucose-1-dehydrogenase (*gdc*) [62]. Based on our results, we identified the *gdc*-encoding gene and the *pqqABCDEF* operon. These genes may play essential roles in the inorganic phosphate dissolution activity of the two strains. Also, they can endorse the previous experimental results, in particular those of S11P1, where it showed the maximum phosphate solubilization activity [13]. The phosphate-specific transporter (*pst*) system of *Bacillus subtilis* has been reported to serves as a high-affinity, low-velocity, and free inorganic phosphate transporter [63]. *Pst* activity is mainly controlled by the *Pho* operon, which is one of the most sensitive and efficient bacterial regulatory mechanisms [64]. The presence of genes encoding *pstACS* permeases and the phosphate signaling complex *phoBHU* in the S11P1 and S12S4 genomes might be pivotal for inorganic phosphate uptake in response to phosphate deficiency. Moreover, the two strains could regulate phosphate homeostasis via polyphosphate kinases (*ppk1* and *ppk2*) either by the accumulation or degradation of polyphosphate. This mechanism can maintain sufficient inorganic phosphate uptake despite the influence of the extracellular environment [65].

Nitrogen is considered one of the most important macronutrients for plant growth and a major limiting factor for crop production [66]. Biological nitrogen fixation, exclusively performed by diazotrophic bacteria, is a process in which atmospheric nitrogen is converted to plant-assimilable forms, such as ammonia and nitrite [67]. The nitrogenase complex, which is responsible for nitrogen fixation, is encoded by the *nif* operon and comprises 20 genes [68]. In this study, 17 genes nitrogenase-encoding genes were identified in the S11P1 genome. The *nifHDK* gene cluster encodes Mo-nitrogenase, which is responsible for nitrogen fixation, whereas *nifBENX* and *nifV* are involved in synthesis and maturation of the FeMo cofactor, respectively [69]. In addition, both strains were predicted to harbor *nirBD* genes encoding nitrite reductases, which are involved in the main reaction of nitrate reduction to ammonium in *Pseudomonas putida* [70], and nitrogen metabolism in *Pseudomonas* sp. XS-18 [71].

Sulfur-oxidizing bacteria have been shown to improve soil fertility by oxidizing elemental sulfur or thiosulfate to plant-available sulfate [72]. Based on our functional annotation, both strains were found to encode enzymes for the metabolism of alkanesulfonates, which are considered a major organosulfur compounds in agricultural soils [73]. The two genomes encode aliphatic sulfonate transporters (*ssuBC*), which transport extracellular alkanesulfonates into the cell for subsequent conversion to sulfite under the action of alkanesulfonate monooxygenase (*ssuD*) and NADPH-dependent FMN reductase (*ssuE*). Moreover, cysteine synthase (*cysK*), encoded by the two strains, was found to catalyze the biosynthesis of cysteine under sulfur-limiting conditions [74]. In addition, the genes *cysCIJN* and 3-mercaptopyruvate sulfurtransferase (*sseA*), identified in S11P1, have been associated with the biosynthesis of hydrogen sulfide (H2S) [75, 76].

Root exudates are the first line of communication between roots and beneficial bacteria [77]. According to our results, the two strains were found to encode a series of genes for chemotaxis and quorum sensing, which are required for rhizosphere and rhizoplane colonization [78]. The presence of the CheAWZ system for signal transduction and the methyl-accepting chemotaxis protein gene (*mcp*) suggests the strong ability of S11P1 and S12S4 to detect different signals in the rhizosphere. The *mcp* gene encodes multiple transmembrane receptors that are responsible for the detection of ligands and the activation of signaling cascade, leading to root-directed motility [79]. Additionally, both genomes harbor the malate dehydrogenase gene (*mdh*), which is essential for *Pseudomonas fluorescens* WCS365 growth on organic acids during tomato colonization [80]. Moreover, the *xerCD* recombinases annotated in the two genomes were found to be crucial for competitive root colonization [81]. Bacterial motility is regarded as a major trait for effective plant root colonization [82]. The *fli*, *flg*, *flh*, and *pil* operons indicate the swimming, swarming, and twitching motility of the two strains. Swimming behavior is initiated after the binding of *cheY* to the flagellar switch protein film [83], whereas twitching motility is associated with the type IV pili protein and prepilin peptidases [84]. In addition, swarming motility has been suggested as a major migration method for tomato root colonization by *Bacillus subtilis* [85]. Previous studies have highlighted the role of the quorum sensing signals in root colonization and induced systemic resistance [86]. The presence of genes encoding quorum-sensing proteins, especially in the S11P1 genome, indicates its ability to mediate plant-microbe and cell-to-cell communication in the rhizosphere.

The biocontrol activity of PGPR can be achieved via various mechanisms, including the production of bacteriocins, lytic enzymes, siderophores, volatile organic compounds, and the induction of systemic resistance [87]. In this study, genomic evidences for all the above-mentioned mechanisms were found in both genomes. These comprise genes for the biosynthesis of antimicrobial and antifungal compounds, such as cobalamin, phenazine, colicin, surfactin, and rhamnolipid. Additionally, the presence of genes responsible for proteolytic, cellulolytic, and chitinolytic activities can promote plants growth by suppressing soil-borne pathogens and maintaining soil ecology and fertility [88]. Moreover, genes encoding systemic resistance inducers such as VOCs have been found in both genomes. It has been shown that VOCs released from *Bacillus* spp. constitute a novel determinants of systemic resistance elicitation in *Arabidopsis* [89]. According to the antiSMASH analysis, BGCs for microcin E492 and microsclerodermin were predicted in the S11P1 and S12S4 genomes, respectively. Microcin E492 is a low-molecular-weight pore-forming bacteriocin that suppresses a broad range of *Enterobacteriaceae* species including *Escherichia*, *Salmonella*, *Enterobacter*, and *Klebsiella* spp [90]. Microsclerodermins are a family of unsual peptides with very high antifungal activities against many pathogenic fungi, including *Candida glabrata*, *Botrytis cinerea*, *Fusarium oxysporum*, *Pyricularia oryzae*, and *Aspergillus fumigatus* [91, 92]. The occurrence of the microsclerodermin gene cluster in the S12S4 genome corroborates the in vitro antagonistic assay results, where S12S4 showed 66.7% inhibition of radial growth of *F. oxysporum* [13]. This may favor S12S4 as a natural biopesticide for managing corm rot disease caused by *F. oxysporum*, which is considered the most devastating disease affecting saffron crops [93].

Abiotic stress negatively affects plant growth and causes large crop yield losses worldwide [94]. The use of stress-mitigating PGPR is an emerging strategy for improving crop quality and yield under adverse environmental conditions [95]. The S11P1 and S12S4 genomes harbor numerous genes involved in environmental stress adaptation. These include genes encoding osmolytes such as trehalose, choline, and proline transporters, which are essential for salt stress tolerance [96]. Additionally, we noted the presence of genes involved in the biosynthesis and transport of spermidine and putrescine, which are associated with lateral root development, balancing ethylene levels, mitigating salinity and drought [97]. The presence of these genes indicates the potential of S11P1 and S12S4 for host habitat adaptation under diverse environmental conditions.

Pan-genome analysis aims to assess the global gene repertoire of a given group of phylogenetically related organisms [98]. The present study predicted an open pan-genome structure for both S11P1 and S12S4. We noted that pan-genome analysis of S12S4 resulted in a lower fraction of core genes compared to the accessory and unique fractions. This observation is in agreement with that reported from a previous pan-genome analysis of all available genomes of *Variovorax* species, and might be related to genome evolution events such as mutation, gene rearrangement, and lateral gene transfer [99]. The attribution of COG functional categories to the pan-genome fractions showed that higher portions of core, accessory, and unique genes were involved in the metabolism and transport of carbohydrates and amino acids. In a previous study, *Bacillus subtilis* inhabiting soil was found to harbor a higher number of genes for carbohydrate metabolism and transport compared to other *Bacillus* spp [100]. In addition, Barret et al. reported that the genes related to rhizosphere adaptation and PGPR-plant interactions and are mainly involved in central metabolism, detoxification, and stress response [101]. Therefore, we suggest that the presence of these gene categories may be advantageous for the metabolic adaptation of S11P1 and S12S4 to root exudates and other plant-derived substrates in the rhizosphere.

## Conclusions

The present study supports, extends, and discusses the molecular basis of the observed plant-beneficial properties of S11P1 and S12S4. Taxonomic investigations placed S11P1 close to the recently described species *R*. *perminowiae* and suggested S12S4 as a potential new species of *Variovorax* genus. Based on our taxonomic investigations, four MLSA markers (16 S rRNA, *gyrB*, *rpoD*, and *rpoB*) were found to be accurate and reliable for describing the phylogenetic position of the two studied strains and were proposed as good MLSA markers for phylogenetic characterization within the *Rahnella* and *Variovorax* genera. Comprehensive genome analyses have predicted and identified the key genes involved in plant growth promotion, biocontrol, and abiotic stress resistance. Comparative genomic analysis of S11P1 and S12S4 with their closest corresponding strains revealed rich core, accessory, and unique genomes, highlighting their ability to adapt and colonize the rhizosphere habitat. To the best of our knowledge, this is the first genomic analysis of PGPR strains isolated from the rhizosphere of saffron plant. Our genomic exploration provides useful clues regarding the biofertilizer and biostimulant potential of S11P1 and S12S4 and paves the way for their large-scale application as eco-friendly bioresources for sustainable agriculture.

## Materials and methods

### Strains isolation and screening for PGP activities

S11P1 and S12S4 were previously isolated from the rhizosphere of *Crocus sativus* L., and soil samples were collected from a saffron farm in the Taliouine-Taznakht region in Morocco (30°28′12.997″N, 7°46′22.479″W) [13]. The two strains were screened for plant growth-promoting traits (auxin and siderophore production, phosphate solubilization, nitrogen fixation, acetyl-CoA carboxylase (ACC), and biocontrol activities) and were tested in field trials, as described previously [13].

### DNA extraction, genome sequencing, assembly, and annotation

Genomic DNA extraction, genome sequencing, and assembly were performed as described previously [49]. Briefly, the genomic DNA was extracted from overnight cultures using a QIAamp Genomic DNA Mini Kit (Qiagen, Germany). The sequencing library was prepared using the rapid barcoding sequencing kit (SQK-RBK004) and the whole genomes sequencing was performed using Nanopore technology. Raw reads were assembled with Canu v.2.2 [102] and polished using Racon v.1.5.0 [103]. The annotation was performed using the NCBI Prokaryotic Genome Annotation Pipeline (PGAP) v.6.2 [104] and Prokka v.1.14.6 [105].

### Identification, phylogenetic, and phylogenomic analyses

The type Strain Genome Server (TYGS) [106] was used for whole genome-based identification of S11P1 and S12S4, which were assigned to *Rahnella perminowiae* and *Variovorax* sp., respectively. The taxonomic positions were determined by multilocus sequence analysis (MLSA) based on four housekeeping genes (16 S rRNA, *gyrB*, *rpoD*, and *rpoB*) retrieved from all publicly available type strain sequences of *Rahnella* and *Variovorax* species. Subsequently, the core genomes of S11P1, S12S4, and their closest corresponding type strains were extracted using Roary v.3.13.0 [107] and used to construct maximum likelihood phylogenomic trees. Alignment of housekeeping genes and core genomes was performed using Mafft v.7.271 [108] and poorly aligned regions were removed using Gblocks v.0.91b [109]. The trees were built with IQ-TREE v.2.2.0.3 [110] using TIM + F + I and GTR + F + I + I + R6 as substitution models for S11P1 and S12S4, respectively. The trees were processed using interactive tree of life (iTOL) v.6.7 [111]. Average nucleotide identity (ANI) and digital DNA-DNA hybridization (dDDH) values were calculated using Pyani v. 0.2.9 [112], and the Genome-to-Genome Distance Calculator (GGDC) v.2.1, (formula 2) [113], respectively. All the strains used for the MLSA and the phylogenomic studies of S11P1 and S12S4 are provided in Supplementary Table S1.

### Genomes functional analysis

The Annotation files of the S11P1 and S12S4 genomes were manually mined for genes associated with plant growth promotion and biocontrol attributes. The collected genes were summarized in a schematic overview created using BioRender (www.biorender.com).

### Biosynthetic gene clusters prediction

Biosynthetic gene clusters (BGCs) were predicted using the Antibiotics and Secondary Metabolites Analysis Shell (AntiSMASH) v.6.0 web server with default parameters [114].

### Comparative genome analysis

All publicly available genome sequences of *R*. *perminowiae* along with the genome sequences of *Variovorax* species sharing ANI values greater than or equal to 89% with S12S4 were selected for pan-genome analysis of S11P1 and S12S4, respectively (Supplementary Table S2). The pan-genome analysis was conducted using Roary v.3.13.0 [107] and the post-processing of pan-genome results was performed through Pagoo tool v.0.3.1.7 [115]. Heap’s law alpha values were estimated using the R package micropan v.2.1 [116] with 100 permutations, and circular maps were generated using the BLAST Ring Image Generator (BRIG) v.0.95 [117]. Furthermore, core, accessory, and unique genomes fractions of S11P1 and S12S4 were fetched and annotated by RPS-BLAST against the Clusters of Orthologous Genes (COG) database using COGclassifier v.1.0.5 [118].

### Electronic supplementary material

Below is the link to the electronic supplementary material.

**Supplementary Material 1:** List of strains used for MLSA and phylogenomic studies of S11P1 and S12S4

**Supplementary Material 2:** List of strains used for the pangenome analysis of Rahnella perminowiae S11P1 and S12S4

**Supplementary Material 3:** Pairwise ANI values and dDDH among S12S4 and all the publicly available type strain sequences of Variovorax species

**Supplementary Material 4:** Secondary metabolites gene clusters predicted in the S11P1 and S12S4 genomes

**Supplementary Material 5:** Genes involved in PGP traits, biocontrol and abiotic stress response predicted for S11P1 and S12S4

**Supplementary Material 6**

## Data Availability

The genome assemblies of *Rahnella perminowiae* S11P1 and *Variovorax* sp. S12S4 are publicly available in NCBI GenBank database under the accession numbers JALMGI000000000 and JALPKR000000000, respectively. BioProject accession number PRJNA827450.

## Abbreviations

ACC: 1-aminocyclopropane-1-carboxylate
ANI: Average Nucleotide Identity
BGCs: Biosynthetic gene clusters
BLAST: Basic Local Alignment Search Tool
COG: Clusters of Orthologous Genes
C-P lyase: Carbon-phosphorus Lyase
dDDH: Digital DNA-DNA Hybridization
IAA: Indole-3-acetic acid
IAM: Indole-3-acetamide
IAN: Indole-3-acetonitrile
IpyA: Indole-3-pyruvate
MLSA: Multilocus sequence analysis
PGPR: Plant Growth Promoting Rhizobacteria
TAM: Tryptamine
TOL: Indole-3-ethanol
VOGs: Volatile organic compounds
