# Supplementary figures and images for: Functional analysis and comparative genomics of Rahnella perminowiae S11P1 and Variovorax sp. S12S4, two plant growth-promoting rhizobacteria isolated from Crocus sativus L. (saffron) rhizosphere
Source: BMC Genomics. 2024 Mar 18;25:289. doi: 10.1186/s12864-024-10088-6 (PMC10946135; doi:10.1186/s12864-024-10088-6)

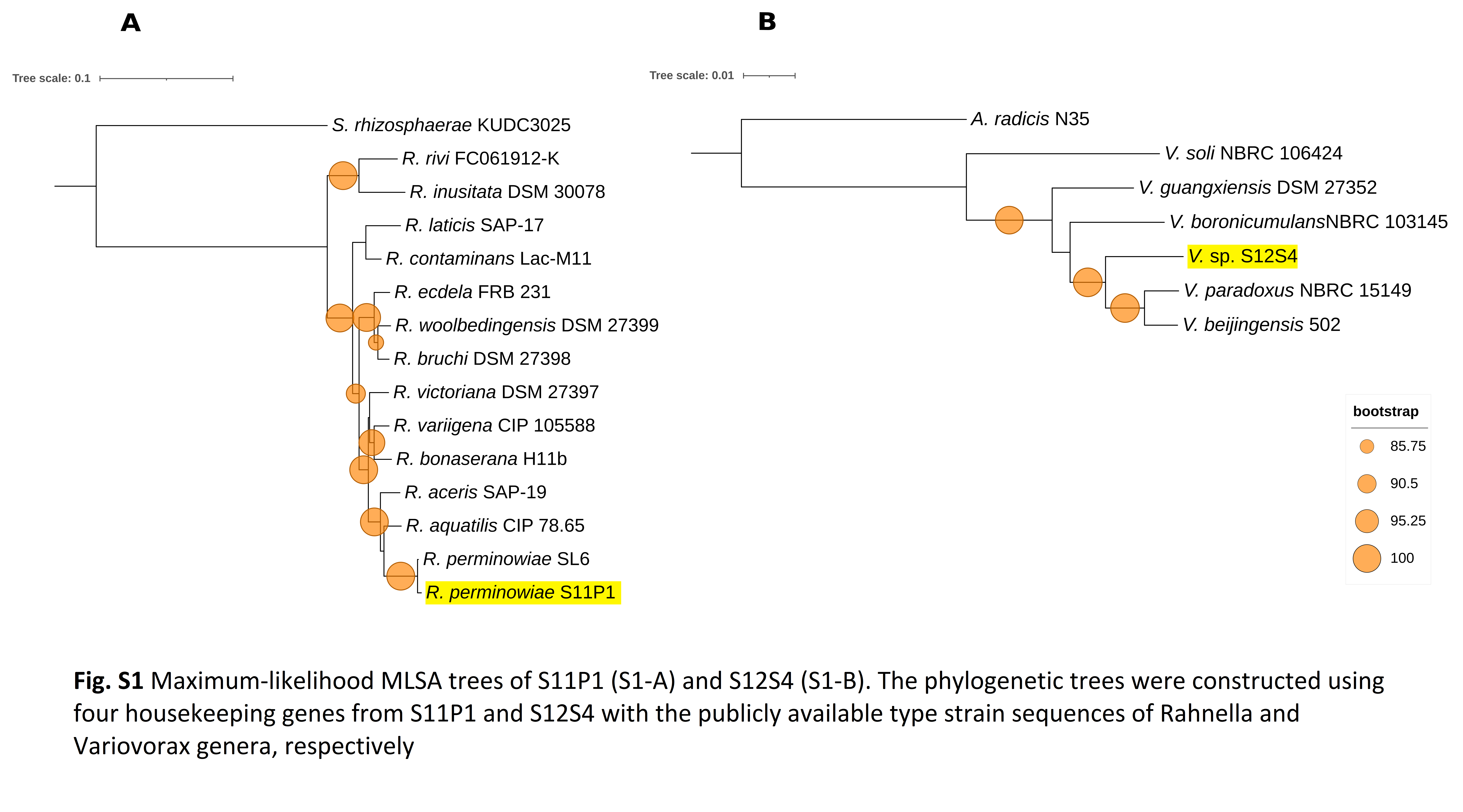

Supplement: Supplementary file 6 — Supplementary Material 6 [file 12864_2024_10088_MOESM6_ESM.tif]
