# Supplementary material for: Functional analysis and comparative genomics of Rahnella perminowiae S11P1 and Variovorax sp. S12S4, two plant growth-promoting rhizobacteria isolated from Crocus sativus L. (saffron) rhizosphere
Source: BMC Genomics. 2024 Mar 18;25:289. doi: 10.1186/s12864-024-10088-6 (PMC10946135; doi:10.1186/s12864-024-10088-6)
Supplement: Supplementary file 7 [file 12864_2024_10088_MOESM4_ESM.odt]

Table S5. Secondary metabolites gene clusters predicted in the S11P1 and S12S4 genomes

| Strain | Region type | Most similar known cluster | Similarity (%) | Core biosynthetic genes/product |
| --- | --- | --- | --- | --- |
| Rahnella perminowiae S11P1 | Betalactone | - | - | leuA, FAA1 |
| Hserlactone | [microcin E492](https://mibig.secondarymetabolites.org/go/BGC0000586/1) | 12% | GNAT |
| NRPS-independent-siderophore | [desferrioxamine E](https://mibig.secondarymetabolites.org/go/BGC0001572/1) | 100% | GNAT |
| NRPS,T1PKS (Hybrid) | [crochelin A](https://mibig.secondarymetabolites.org/go/BGC0002001/1) | 8% | NRP synthetase, alnF |
| NRPS-independent-siderophore | Vibrioferrin | 57% | Siderophore biosynthesis protein |
| Hserlactone,phosphonate (Hyrid) | [N-myristoyl-D-asparagine / cis-7-tetradecenoyl-D-asparagine / (R)-N1-((S)-5-oxohexan-2-yl)-2-tetradecanamidosuccinamide](https://mibig.secondarymetabolites.org/go/BGC0000972/1) | 8% | NAT_SF, aepX |
| Thiopeptide | [O-antigen](https://mibig.secondarymetabolites.org/go/BGC0000781/1) | 14% | ycaO, PFL-AE |
| Arylpolyene | [aryl polyenes](https://mibig.secondarymetabolites.org/go/BGC0002008/1) | 77% | KAS |
| RRE-containing | [vibrioferrin](https://mibig.secondarymetabolites.org/go/BGC0000947/1) | 27% | pqqD |
| Variovorax sp. S12S4 | NRP-metallophore, NRPS, T1PKS | variochelin A/variochelin B | 90% | iucD, fkbM_fam, type I PK synthase |
| Arylpolyene | - | - | KAS |
| Redox-cofactor | - | - | pqqC,[ pqqD, ](https://blast.ncbi.nlm.nih.gov/Blast.cgi#alnHdr_MCK8447343)pqqE |
| Resorcinol | - | - | I[ron-containing redox enzyme ](https://blast.ncbi.nlm.nih.gov/Blast.cgi#alnHdr_WP_157613671) |
| Terpene | - | - | hpnC, hpnD, |
| T1PKS,NRPS-like,NRPS (Hybrid) | [microsclerodermin](https://mibig.secondarymetabolites.org/go/BGC0001231/1) | 21% | NRP synthetase |
| Arylpolyene,resorcinol (Hybrid) | [Lipopolysaccharide](https://mibig.secondarymetabolites.org/go/BGC0000774/1) | 5% | KAS, KASIII |

Genes key: 

leuA: 2-isopropylmalate synthase

FAA1: long-chain fatty acid--CoA ligase

alnF: [amino acid adenylation domain-containing protein](https://blast.ncbi.nlm.nih.gov/Blast.cgi#alnHdr_WP_241921827)

PFL-AE: pyruvate formate-lyase activating enzyme

GNAT: GNAT family N-acetyltransferase

NRP synthetase: non-ribosomal peptide synthetase

Type I PK synthase: type I polyketide synthase

KAS: beta-ketoacyl synthase

KASIII: beta-ketoacyl-ACP synthase III

pqqC: pyrroloquinoline-quinone synthase PqqC

pqqD: pyrroloquinoline-quinone biosynthesis peptide chaperons PqqD

pqqE: pyrroloquinoline-quinone biosynthesis protein PqqE

Ion-containing redox enzyme: Ion-containing redox enzyme family protein

DUF692: DUF692 family protein

iucD: lysine N(6)-hydroxylase

fkbM_fam: Amino acid adenylation domain-containing protein

HpnC: qualene synthase

HpnD: presqualene diphosphate synthase

NAT_SF: N-acyl-L-homoserine lactone synthetase

aepX: phosphoenolpyruvate mutase

YcaO: YcaO cyclodehydratase, ATP-ad Mg2+-binding; cl19253
